# A Sociology of Plasma Proteins – A Technocrat's Perspective

**DOI:** 10.1177/27551938241264764

**Published:** 2024-08-14

**Authors:** Albert Farrugia

**Affiliations:** 1 School of Surgery, University of Western Australia Medical School, The University of Western Australia (M509), Perth, Australia

**Keywords:** blood donation, plasma products, ethics

## Abstract

This commentary addresses the article “Toward a Sociology of Plasma Products” by Holloway and Grundy in this issue of the *International Journal of Social Determinants of Health and Health Services*. The program of research proposed by the authors positioning the medico-industrial field of plasma products within a sociological context is supported, this being an endeavor which has not been attempted previously. I seek to augment Holloway and Grundy's proposed approach through some additional insights which are the result of over forty years of personal commitment in the field. Holloway and Grundy's proposed areas of engagement involving the products, the recipients, the donors and the governance of the systems binding these together is widened through an examination of additional technological factors that have shaped the field. These factors include the influence of the medical industry, the role of patient groups, the continuing controversy on the sourcing of plasma raw material, and the roles of different governance models. Converging these factors with Holloway and Grundy's proposed program should enhance its capacity to develop a framework for understanding the dynamics within this complex and unique sector. The concepts developed in both articles will assist stakeholders to develop a societal framework for the provision of these essential medicines.

In this issue of *International Journal of Social Determinants of Health and Health Services*, Holloway and Grundy propose in their article “Toward a Sociology of Plasma Proteins” a comprehensive program of work to widen the scholarly landscape around a class of medicines—plasma-derived medicinal products (PDMPs)—through including a sociological dimension and synthesizing a model integrating the players interacting in their provision. Their review touches upon most of the issues that engage these players, most of whom are active workers in the field and, as hinted by Holloway and Grundy, have a perspective which may be restricted and unidimensional. This commentary is offered by one such player, currently exiting the world of work, but influenced through having participated in most of the sectors within the field, not least of which is the experience of a patient using these medicines.^1^ I will assess certain aspects of Holloway and Grundy's paper, attempt to augment some of its techno-specific comments, and offer further areas for their and other sociologists’ attention, aiming to further a sociology of PDMPs.

## Plasma-Derived Medicinal Products: A Unique Class of Medicines (?)

PDMPs are unusual, if not unique, because they belong to a class of therapeutics derived from human tissue. Other such therapeutics include organs, cell- and tissue-based therapies involved in regenerative medicine, and gene therapies. Although some of these therapeutics are provided through public health-based processes, they are all subject to increasing levels of commercialization,^2^ with the recently commercialized gene therapy for hemophilia B being priced at $US 3.5 million.^3^ Hence, it is not the particular raw material in the form of the recently specified substances of human origin (SOHOs),^4^ that makes PDMPs unique. Neither is the commercialization aspect, which permeates across the whole SOHO landscape. It is the case that the multiplicity of products and stakeholders generated by the aspects explored by Holloway and Grundy impose characteristics that are less evident in other SOHOs and other therapeutic groups, albeit to an increasingly lower degree. Some of these aspects will now be assessed through furthering the lines of inquiry proposed by Holloway and Grundy.

## Examining the Promise of Plasma Products: Has the Promise Delivered?

The development of the concept of evidence-based medicine (EBM) has been pivotal in the current paradigm for therapeutic provision,^5^ yet it appears to have escaped the notice of Holloway and Grundy. Their suggestion that more data is required on the appropriate use of immunoglobulins (IG), the standardization of guidelines, and so on, suggests doubts on the efficacy of these PDMPs. EBM underpins the provision of IG in the established economies, forming the basis of regulatory approval, a necessary prelude to market access. This process specified which clinical conditions can be treated with IG and which doses are recommended as shaped by mandatory clinical trials. This then feeds into the relevant guidelines, which influence usage in the main clinical landscapes globally. There is little variation between these guidelines, and it is difficult to endorse the allocation of effort in this specific aspect as considered necessary by Holloway and Grundy. The usage of IG is predominantly in the highest level of evidence-based clinical indications, and it is noteworthy that two countries that control the provision of IG through these guidelines—Australia and Canada—are both social-market economies with the highest IG usage per capita globally. Evidence-guided clinical practice increases, not curtails, IG usage, in contrast to the tentative suggestions of Holloway and Grundy.

The fractional nature of the manufacturing process for PDMPs has provided one of the main strengths of the plasma industry as it provides, theoretically, access to the multitude of proteins in plasma. The economic viability of the industry depends on its ability to extract and market as many proteins as possible, yet, since its inception, the industry has relied on a handful of products representing a small fraction of the therapeutic proteins in plasma. This handful has shrunk further with the replacement of blood coagulation proteins, previously PDMPs, by disruptive technologies,^6^ and the viability of the industry is heavily dependent on IG, itself pressured by disruptive alternatives.^7^ The emergence of these therapies has the potential to alter radically the IG therapeutic landscape, and shift the costs of IG and the price of the product to a narrow band of indications currently not threated by these new technologies, such as immunodeficiency. One could consider including in Holloway and Grundy's program an assessment of why the industry has neglected to develop products for the range of rare conditions, such as congenital deficiencies of prothrombin, coagulation factor V, and others for which purification methods exist but have not been pursued by the industry, possibly for commercial reasons.

## Locating the Recipient in a Sociocultural Context

Holloway and Grundy imply that patients, through their associations, are influenced unduly by the plasma industry that manufactures IG “and could be invested in their expanded use.” Although rich in anecdotal discourse, there is little documented research on this issue of the “capture” of organizations of patients dependent on PDMPs by the plasma industry. There is ample evidence of industry support for patient organizations not supported by the state authorities, as, apart from a modest input from private donations, no other source of funding is visible. Without this industry funding, patient organizations—and all the work they do for their constituency—would not exist. In some environments, these relationships are a source of concern.^8^ Certain activities, particularly the continuous conference circuit with its industry-sponsored and never-changing patient Key Opinion Leaders (“KOLs,”) can be easily rationalized without damaging the necessary networking activities. Direct endorsement of a particular product brand especially should be avoided.

The centrality of the patient is now accepted as an essential adjunct to the EBM process. In order to demonstrate *effectiveness* as well as *efficacy* based on clinical trials, assessments of health-related quality of life (HrQoL) are part of most clinical trials in order to understand patients’ perceptions of how treatments affect them. The particular social perceptions of people with a lifelong rare disease have been researched, and knowledge gaps identified.^9,10^ The possible influence of pharmaceutical companies on this research has also been noted.^10^ In the context of assessing health technologies of effectiveness, we have commented on the limitations of HrQoL instruments, and the possible influence of the “disability paradox,” which influences the perceptions of patients with rare diseases.^11,12^ These factors demand consideration if such patients are not to be disadvantaged. Holloway and Grundy's focus on the differential positioning of high and low to middle-income countries (HIC versus LMIC) is appreciated when the experience of patients with rare diseases in these environments is studied^13,14^; in these circumstances, there is little evidence of a “disability paradox.”

## Engaging the Donor as an Actor in the Blood System

Holloway and Grundy allude to the longstanding controversy regarding paid versus unpaid donation, a debate revisited recently through the European Commission's development of a regulation on SOHOs.^4^ There is little doubt that the primary motivation for the population contributing two thirds of the global supply of PDMPs is financial compensation, underpinned by severe economic disadvantage.^15^ Against this reality is the continued failure of the publicly owned blood and plasma collection system globally to generate sufficient plasma to meet clinical needs, as a result of the reluctance to invest in the relevant infrastructure. A considerable literature has evolved on the motivation of different types of blood and plasma donors, and we have proposed aligning these different populations to Maslow's Hierarchy of Needs.^16^ The separate demographics of paid and unpaid donors do not overlap significantly, and the motivations of the latter, founded on issues of prestige, access to health checks, days off work, and so on, along with altruism and variants thereof, are also fundamental in their decision to donate. We draw Holloway and Grundy's attention to data showing that racial minorities are overrepresented, not underrepresented, in the paid plasma demographic due to the economic disparities noted above.^17^

Holloway and Grundy's identification of the role of plasma center staff is well taken, with semi-anecdotal evidence of exploitative work conditions^18^ augmenting the confronting landscape that the entirety of the paid plasma sector presents to the field of PDMPs. Their concerns regarding the restrictions placed on other groups, historically associated with higher risks of infectious disease transmission, may benefit from a recognition that such risks persist in, for example, the area of pre-exposure prophylaxis (PrEP) for HIV infection,^19^ justifying deferral measures in the interest of patient safety.

## Considering the Role of Governance

The governance of the blood system has been shaped by the extent to which it has been centralized to one responsible entity or diffused across multiple agencies. In most of the European states, the system in place devolved to individual hospitals, and therefore is subject to their budgetary control. This is exemplified by Italy and Spain. In other countries, notably Australia and Canada, a system centralized under one responsible agency collects blood and plasma, directly or indirectly funded by the state.^20^ In some instances, governments control the system through an agency charged with managing the whole landscape of procurement, manufacture, and distribution, as exemplified by the Australian National Blood Authority (www.nba.gov.au). The factors that shape efficiency in the procurement of plasma are independent of the model in place, as Australia and their centralized system^21^ and the Czech Republic with a totally decentralized system^22^ comprise the two top collectors of plasma from unpaid donors, and, in the case of the Czech Republic, of paid plasma as well. In this latter jurisdiction there is no link between plasma collection and the return of PDMPs to the public system, a state of affairs which lends itself to research on the views of Czech donors and patients, who are clearly underserved in IG, on the particular governance model in their country.

## Conclusion

Clearly, many of the issues mentioned in this commentary can be integrated within the sociological framework proposed by Holloway and Grundy. Interrogation of such a framework with further research questions will enhance its applicability as a tool for the various stakeholders in this complex landscape. A happy convergence between technocrats and sociologists should generate a productive synthesis and allow further work to be done in this complex area.
